# Function of the Chondrocyte PI-3 Kinase-Akt Signaling Pathway is Stimulus Dependent

**DOI:** 10.1016/j.joca.2015.01.014

**Published:** 2015-02-03

**Authors:** Meredith A. Greene, Richard F. Loeser

**Affiliations:** * Department of Internal Medicine, Section on Molecular Medicine, Wake Forest University School of Medicine, Winston-Salem, North Carolina; † Thurston Arthritis Research Center, University of North Carolina, Chapel Hill, North Carolina

**Keywords:** PI-3 kinase, chondrocyte, cell signaling, matrix metalloproteinase-13

## Abstract

**OBJECTIVE:**

The PI-3 kinase-Akt pathway plays a role in cartilage anabolic as well as catabolic processes in response to activation by insulin-like growth factor-1 (IGF-1) and the pro-inflammatory cytokines interleukin-1β (IL-1β) and oncostatin M (OSM). The goal of this study was to determine how PI-3 kinase-Akt signaling regulates these seemingly opposing functions.

**DESIGN:**

Monolayer cultures of primary human articular chondrocytes were treated with IGF-1, IL-1β, OSM, or the combination of IL-1β and OSM in time course experiments. Activation of signaling proteins and MMP production were measured by immunoblotting. Cells were pre-treated with chemical inhibitors to block MAP kinases, PI-3 kinase, or JAK/STAT pathway activation. Constitutively active Akt1 and Akt3 were expressed to study stimulus-independent activation of Akt.

**RESULTS:**

IGF-1, OSM, and the combination of IL-1β and OSM but not IL-1β alone, stimulated phosphorylation of Akt which was sustained longer with IGF-1. IL-1β plus OSM, but not IGF-1, increased chondrocyte MMP-13 production which was inhibited with either a general PI-3 kinase inhibitor or specific inhibition of the PI-3 kinase-γ isoform. Akt1 or Akt3 activity alone was not sufficient to increase production of MMP-13. IL-1β/OSM induced MMP-13 production required activation of the MAP kinases, JNK and p38, as well as the JAK-STAT pathway which were activated by IL-1β plus OSM but not by IGF-1.

**CONCLUSIONS:**

The chondrocyte integrates signals from the PI-3 kinase-Akt pathway with signals from MAP kinases and the JAK-STAT pathway to allow for a differential response to a pro-anabolic (IGF-1) and a pro-catabolic (IL-1β plus OSM) stimulus.

## Introduction

The activity of intracellular signaling pathways in chondrocytes is regulated by soluble mediators and by changes in the extracellular matrix (1, 2). Understanding specific signaling pathways that play a role in osteoarthritis (OA) is of interest since these pathways could serve as therapeutic targets. Major pathways that have been shown to be active in OA chondrocytes include the mitogen activated protein (MAP) kinase family, the Wnts, Smads, the JAK-STAT pathway, toll-like receptor pathways, and the NF-κB pathway (1, 3). These pathways mediate both anabolic and catabolic functions in cartilage and likely work in concert to determine the overall balance of matrix synthesis and degradation.

There is some controversy over the potential role of the chondrocyte PI-3 kinase-Akt pathway in OA. Stimulation of this pathway by insulin-like growth factor 1 (IGF-1) has been shown to promote chondrocyte survival as well as proteoglycan and collagen synthesis (4–6). However, other studies have shown that the PI-3 kinase-Akt pathway is also activated by inflammatory cytokines, such as interleukin-1β (IL-1β) and oncostatin M (OSM), resulting in increased production of MMPs, including the collagenase MMP-13, resulting in cartilage matrix loss (7–9). Additionally, Akt isoforms 1 and 3 have been shown to be necessary for the IL-1β/OSM stimulated MMP-13 production (7). These findings describing the role of the PI-3 kinase-Akt pathway in MMP-13 production have led to the suggestion that this pathway would be an effective target in the treatment of OA (10).

In order to better understand the potential role of the PI-3 kinase-Akt pathway in regulating anabolic and catabolic processes in cartilage, we compared chondrocyte signaling in response to stimulation with the anabolic factor IGF-1 and the catabolic mediators IL-1β and OSM. In chondrocytes, IGF-1 signals through the PI-3 kinase-Akt pathway to promote cell survival and matrix synthesis (2, 3). IL-1β increases MMP production through activation of the MAP kinase pathways (ERK, JNK, and p38) (11). OSM, a member of the IL-6 family, increases MMP production through activation of the JAK/STAT pathway (12). The combination of IL-1β and OSM is a more potent stimulus of MMP production and cartilage matrix destruction than either cytokine alone and thought to be relevant to promotion of cartilage loss in arthritis (7, 13).

The response of cells to growth factors and cytokines most often depends on integration of multiple signals that are generated in a time dependent fashion and are subject to positive and negative feedback loops. Here, we focused on the time dependent activation of key nodes in the IGF-1, IL-1β, and OSM pathways to examine how these stimuli, which share activation of the PI-3 kinase-Akt pathway, result in either pro-anabolic activity, in the case of IGF-1, or pro-catabolic activity, in the case of IL-1β and OSM. We confirm previously published results that both IGF-1 treatment and IL-1β/OSM co-treatment result in Akt phosphorylation, and inhibition of PI-3 kinase blocks IL-1β/OSM stimulated MMP-13 production. Additionally, we show that active Akt by itself is not sufficient for MMP-13 production and that the difference between IGF-1 and IL-1β/OSM in stimulation of MMP-13 is due to differential activation of MAP kinases and STAT3. These findings suggest that MAP kinase or JAK-STAT pathways would be more appropriate as a therapeutic target given the pro-anabolic and cell survival role of Akt.

## Experimental

### Reagents

Chemical inhibitors were purchased from Calbiochem (LY294002, PD98059, SB203580), Sigma (SP600125), and SelleckBiochem (A66, TGX-221, CAL-101, AS-252424, Ruxolitinib). The doses used and specific targets for each inhibitor are summarized in Table 1. Recombinant proteins were purchased from Austral Biological (IGF-1) and R&D Systems (IL-1β, OSM, IL-6, sIL-6R). Antibodies were purchased from Cell Signaling Technology (p-Akt S473, T-Akt, p-ERK T202/Y204, T-ERK, p-JNK T183/Y185, T-JNK, p-p38 T180/Y182, T-p38), Santa Cruz Biotechnology (p-STAT3 Y705, T-STAT3), and Abcam (MMP-13, MMP-2).

### Chondrocyte Isolation and Cell Culture

Chondrocytes were isolated from normal human ankle (talar) or knee cartilage obtained from tissue donors or osteoarthritic knee cartilage removed during joint replacement surgery. Normal donor tissue was obtained from deceased tissue donors through the Gift of Hope Organ and Tissue Donor Network (Elmhurst, IL) and Rush University Medical Center (Chicago, IL). OA tissue was removed from the joint at time of knee replacement surgery at Wake Forest Baptist Health (Winston-Salem, NC). Human tissue use was approved by the Institutional Review Boards at Rush University, Wake Forest University School of Medicine and the University of North Carolina at Chapel Hill. Briefly, the cartilage was dissected away from the subchondral bone and digested in Pronase (Calbiochem) for 1 hour at 37°C followed by overnight digestion in Collagenase P (Roche Diagnostics) at 37°C. Cells were cultured in high density monolayers with 10% serum in D-MEM media (Gibco) until confluent and then made serum-free overnight before treatment. Cells were treated with 100 or 50ng/ml IGF-1 (lower dose used for PI-3 kinase isoform inhibition studies), 0.2 or 10ng/ml IL-1β (lower dose used in experiments in Figure 3, but no difference was seen with high dose of IL-1β in combination with OSM, so 10ng/ml was used in all other experiments), or 10ng/ml OSM.

### Lentivirus Infection

Akt1 lentiviral constructs were kindly provided by Dr. Michael Robinson (Children’s Hospital of Philadelphia), and details of the preparation of the lentivirus were previously published by our group (5). Chondrocytes were infected with lentivirus within 72 hours of plating. The lentivirus (25,000 viral particles/ml) was diluted in 10% serum media containing 8mg/ml Polybrene and incubated at 37°C. The media containing lentivirus and Polybrene was replaced after 24 hours. Forty-eight hours post-infection, cells were made serum-free for six hours and then co-stimulated with 10ng/ml IL-1β and 10ng/ml OSM overnight.

### Nucleofection

Wild-type (pCMV5. HA PKBγ) and dominant negative (pCMV5. HA PKBγ T305A/S472A) Akt3 plasmids were kindly provided by Brian Hemmings (Friedrich Miescher Institute, Switzerland). Constitutively active Akt3 plasmid (1236 pcDNA3 Myr HA Akt3) was purchased from Addgene (plasmid #9017).

After isolation and overnight digestion, chondrocytes were plated and allowed to recover for 48–72 hours. Cells were then incubated for 3 hours in collagenase and pronase together at 37°C to digest adhesion proteins and lift the cells from the plate. The cells were rinsed once in 1X Dulbecco’s phosphate buffered saline (DPBS, Lonza), and then resuspended in Human Chondrocyte Nucleofector Solution (Lonza) at a density of 1×10^6^ cells/100µL solution. After combining 100µL cell solution with 2µg of appropriate DNA plasmid, the cells were transferred to an Amaxa cuvette and placed in the Amaxa Nucleofector II. Following nucleofection, cells were placed in 20% serum media and plated in Poly-L-lysine (Sigma) coated tissue culture plates. After overnight incubation in 20% serum media to allow recovery and to promote adherence of nucleofected cells, the media was changed to 10% serum until cells were serum-starved prior to treatment.

### Immunoblotting

Following treatment, high density chondrocyte monolayers were rinsed twice with 1X DPBS and lysed for 30min in lysis buffer containing 20mM Tris-HCl, 150mM NaCl, 1mM Na_2_EDTA, 1mM EGTA, 1% Triton, 2.5mM sodium pyrophosphate, 1mM β-glycerophosphate, 1mM Na_3_VO_4_, 1µg/ml leupeptin (Cell Signaling Technology) with phenylmethanesulfonyl fluoride (PMSF, Sigma) and Phosphatase inhibitor cocktail 2 (Sigma). The cell lysate was centrifuged for 10min at 13,000rpm to remove the insoluble fraction. The protein content of the soluble fraction was quantified using the Pierce Micro BCA kit (Thermo Scientific). Approximately 10µg protein/sample were combined with 2X Laemmli Sample Buffer (Bio-Rad) containing 5% β-mercaptoethanol, boiled for 5min, and loaded on 10% SDS-PAGE gels to run for 90min at 120 volts. The protein was transferred to Whatman nitrocellulose membrane for 90min at 120 volts. Membranes were blocked for 60min in 5% blocking buffer (Bio-Rad) and then incubated with the primary antibody overnight at 4°C. Following primary antibody incubation, membranes were rinsed three times in TBST, incubated with HRP-tagged secondary antibody (Cell Signaling Technology) for 60min, and rinsed three times in TBST. Blots were developed using Amersham ECL reagent and visualized. Protein kinase phosphorylation was measured at indicated time points by immunoblotting cell lysates with phospho-specific antibodies. Blots were stripped and reprobed with antibodies to the total protein as a loading control

For conditioned media, equal volumes of media were run on SDS-PAGE gels and used for immunoblotting for MMP-13. Blots were stripped and probed for MMP-2. The levels of MMP-2 were not altered by the stimuli or inhibitors tested and so were used as a loading control. Immunoblots were quantified by densitometry using ImageJ software.

### RT^2^-qPCR

Following overnight treatment of chondrocytes, the cells were rinsed twice and then lysed in Trizol (Invitrogen). RNA was isolated from the cell lysate using the standard Trizol isolate protocol. Briefly, chloroform was added to the Trizol lysate, and the samples were centrifuged separate phases. The RNA-containing aqueous phase was transferred to a new tube, and isopropanol was added to precipitate the RNA. The RNA pellets were washed once with 70% ethanol, and the RNA was quantified using the UV/VIS spectrophotometer. The reverse transcription reaction was performed using 2µg RNA/sample. RT enzymes and reagents were purchased from Promega. RT^2^-qPCR was performed using SybrGreen Master Mix and gene-specific oligos purchased from Qiagen. Each sample was assayed in three technical replicates.

### ELISA

MMP-13 was quantified in conditioned media using the MMP-13 solid-phase ELISA kit from R&D systems. Samples were diluted in Calibrator Diluent (included in kit) and analyzed following the protocol included in the kit. Each sample was assayed in two technical replicates. MMP-13 content per sample was calculated from the standard curve, corrected for the dilution factor, and then normalized to total protein measured in the cell lysate from each sample.

### Statistical Analysis

Statistical analysis was performed using GraphPad Prism 6 (GraphPad Software, Inc.). Densitometry, qPCR, and ELISA data from at least three independent experiments, each performed using cells from a unique donor (n for each set of experiments is noted in the figure legend where n=the number of independent experiments), were analyzed by one-way analysis of variance (ANOVA), followed by Tukey’s Honest Significant Difference (HSD) post-hoc analysis. Data are presented graphically as individual points, each representing an independent experiment with the mean for each condition. In the figure legends the mean ± standard deviation are provided.

## Results

### Activation of PI-3 Kinase and MAPK signaling pathways by IL-1β, OSM, and IGF-1 is time- and stimulus-dependent

**Figure 1:**
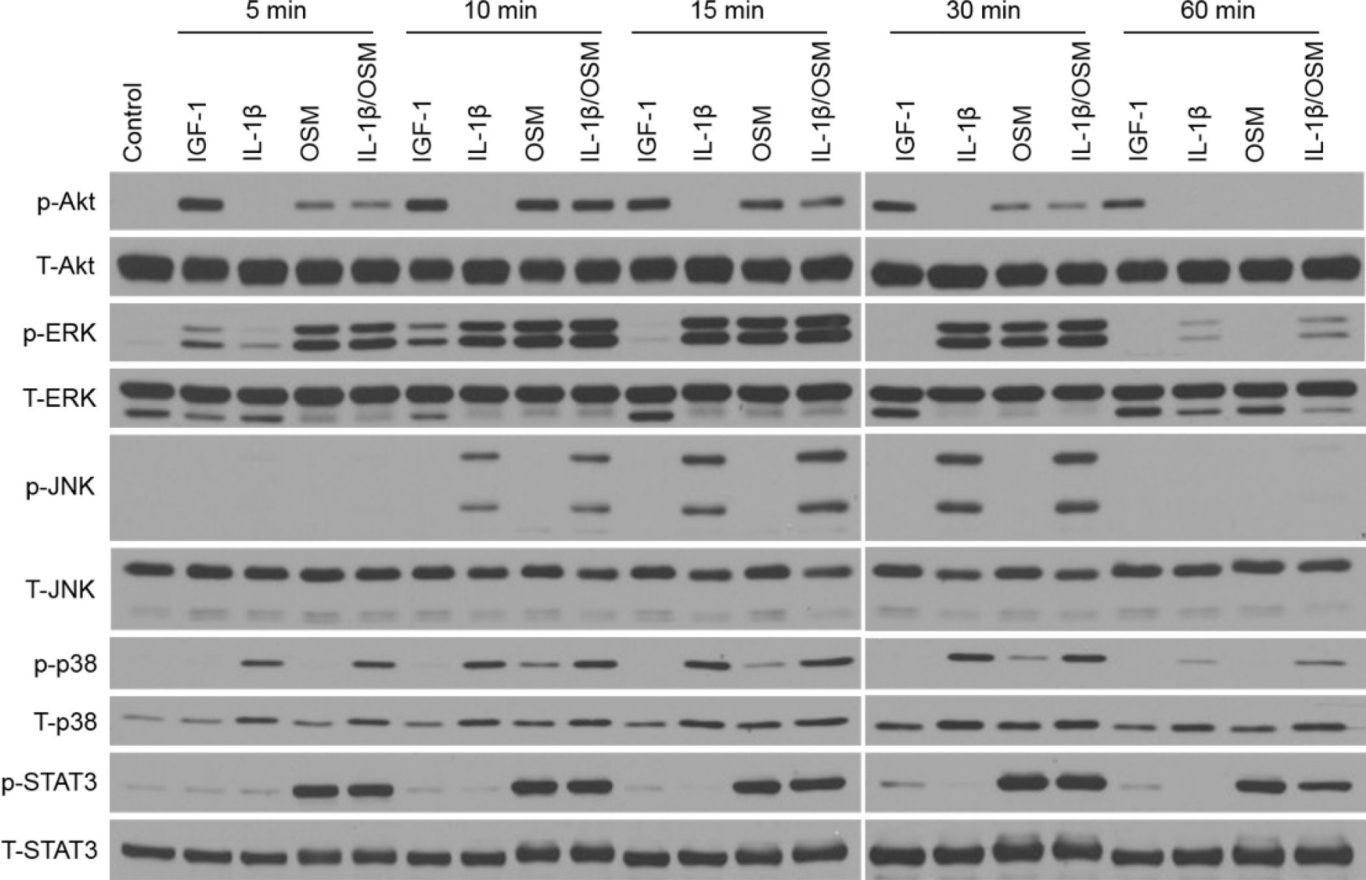
Time course of chondrocyte signaling activated in response to IGF-1, IL-1β, and OSM Normal human chondrocytes were stimulated with IGF (100ng/ml), IL-1β (10ng/ml), OSM (10ng/ml), or IL-1β and OSM together (10ng/ml each) for increasing time points (5–60min). Total cell lysates were immunoblotted for phosphorylated signaling kinases; total kinase blots are shown as loading controls. Representative blots are shown from three independent experiments performed using cells from three different tissue donors.

**Figure 2:**
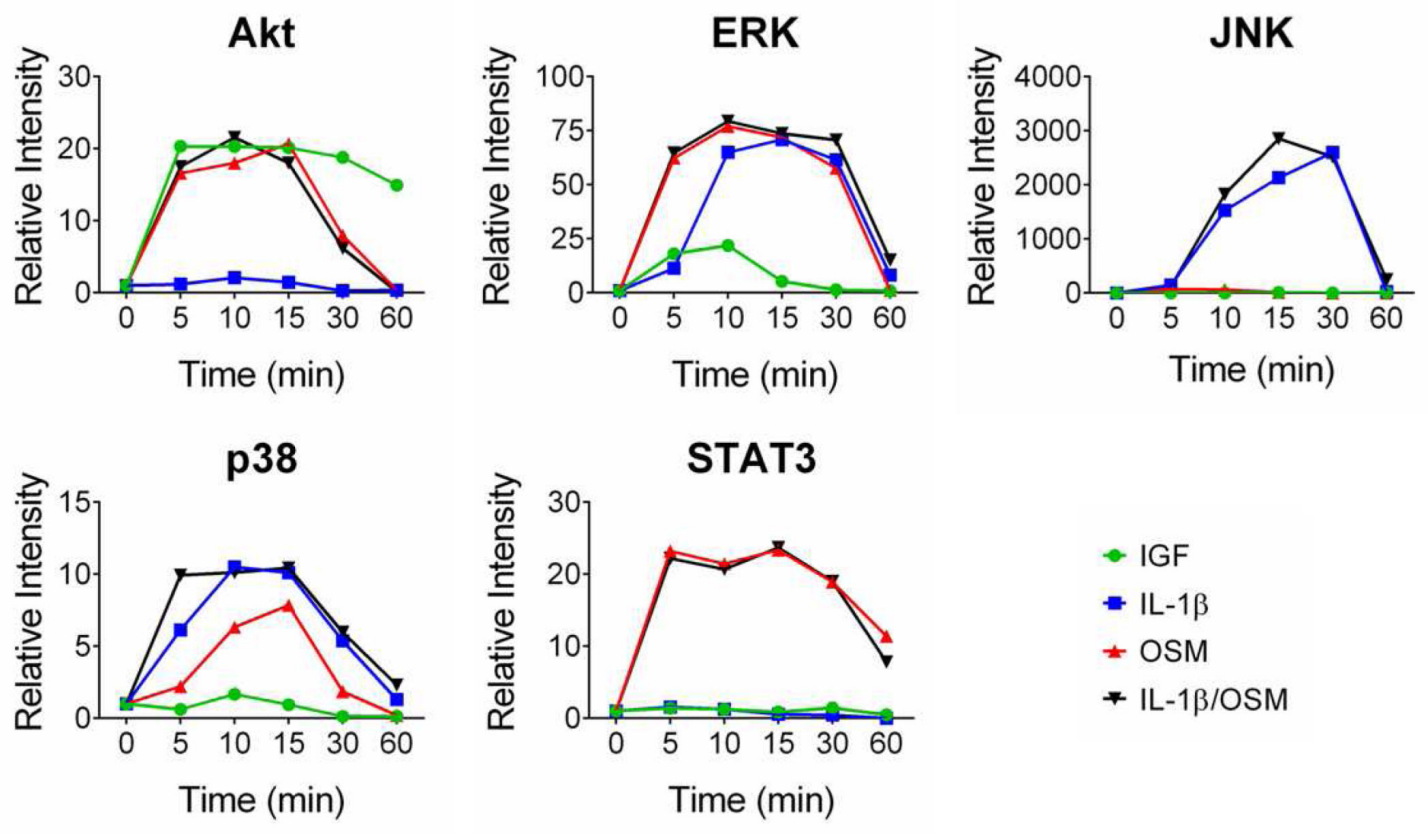
Densitometric analysis of time course of chondrocyte signaling activated in response to IGF-1, IL-1β, and OSM The signal of the phosphorylated kinase was normalized to the total kinase loading control and shown as fold-change from untreated control. Data are shown as the average of three independent experiments performed using cells isolated from three different normal tissue donors.

The downstream effects of cell signaling are dependent on the time required to activate a particular pathway as well as the level of activation and the longevity of the signal. We used a time course analysis to directly compare the anabolic factor IGF-1 with the catabolic factors IL-1β and OSM to examine similarities and differences in the response. Primary articular chondrocytes were treated with IGF-1, IL-1β, OSM, or the combination of IL-1β and OSM at increasing time points from five to 60 minutes and relevant signaling proteins were evaluated for phosphorylation status which, for the chosen proteins, correlates with activation. Representative immunoblots are shown in Figure 1 and densitometric results showing the averages from three independent donors are shown in Figure 2 to illustrate the different patterns in the responses of each signaling protein to a particular stimulus.

The anabolic stimulus IGF-1 at 100ng/ml stimulated Akt phosphorylation which was detectable at 5 minutes and sustained out to 60 minutes. IGF treatment resulted in weak and transient ERK phosphorylation that peaked at 10 minutes and was not detectable after 15 minutes. IGF treatment did not result in detectable phosphorylation of the JNK or p38 MAP kinases or STAT3.

IL-1β alone at 10ng/ml did not stimulate detectable Akt or STAT3 phosphorylation at any of the time points examined. Higher doses of IL-1β (up to 1µg/ml) were also tested and did not stimulate detectable Akt phosphorylation (data not shown). IL-1β stimulated phosphorylation of ERK and JNK, with a slight increase in phosphorylation at 5 minutes followed by peak phosphorylation between 10 and 30 minutes and a decrease in phosphorylation at 60 minutes. IL-1β stimulated phosphorylation of p38 at 5 minutes, followed by maximum phosphorylation from 10–15 minutes with little phosphorylation noted at 60 minutes.

OSM alone at 10ng/ml stimulated phosphorylation of Akt at 5 to 15 minutes that decreased at 30 minutes and was undetectable by 60 minutes. OSM stimulated strong ERK phosphorylation at 5 minutes that was sustained out to 30 minutes but not detectable at 60 minutes. OSM did not stimulate detectable JNK phosphorylation and stimulated weaker phosphorylation of p38 than IL-1β. OSM also stimulated strong phosphorylation of STAT3 at 5 minutes which was sustained out to 30 minutes and began to decrease at 60 minutes. The strong stimulation of p-STAT3 by OSM is expected since OSM is a member of the IL-6 family of cytokines, and IL-6 is a strong stimulator of the JAK/STAT pathway (12).

The combination of IL-1β and OSM stimulated transient phosphorylation of Akt that was slightly stronger than the OSM-induced phosphorylation of Akt; Akt phosphorylation began to decrease at 30 minutes and was not detectable at 60 minutes. IL-1β/OSM stimulated ERK phosphorylation from 5–30 minutes to a level similar to IL-1β alone or OSM alone. IL-1β/OSM stimulated JNK phosphorylation from 10 to 30 minutes (similar to IL-1β alone), and the phosphorylation signal was not detectable at 60 minutes. IL-1β/OSM stimulated phosphorylation of p38 from 5 to 30 minutes. IL-1β/OSM induced strong phosphorylation of STAT3 at 5 minutes, similar to OSM alone, that was sustained out to 30 minutes and began to decrease at 60 minutes.

### PI-3 kinase inhibition blocks IL-1β/OSM stimulation of Akt phosphorylation and MMP-13 production

**Figure 3:**
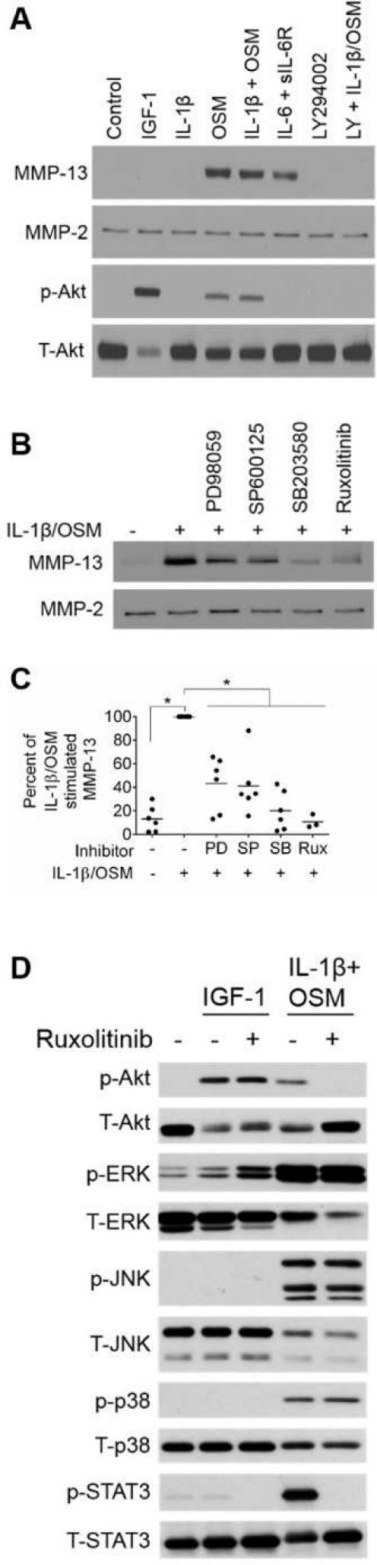
PI-3K, MAPK, and STAT inhibition block IL-1β/OSM induced MMP-13 production **A.** Normal chondrocytes were pre-treated with 5µM LY294002, followed by 30 min stimulation (p-Akt blot) or overnight stimulation (MMP-13 blot). Representative blots are shown (n=3 independent experiments using cells from three different tissue donors). MMP-2 is used as a loading control for MMP-13, and total Akt is shown as a loading control for p-Akt. Strong p-Akt in the IGF-1 stimulated cells reduces the ability of the total antibody to recognize Akt. **B.** Chondrocytes were pre-treated with inhibitors for 30 min and then stimulated with IL-1β/OSM overnight. Conditioned media was collected and immunoblotted for MMP-13. MMP-2 was used as a loading control. **C.** Densitometric analysis of immunoblots from independent experiments in **B** (n=6 independent experiments with cells from six unique tissue donors, controls and MAPK inhibitors; n=3 independent experiments using cells from three unique tissue donors, Ruxolitinib). Data are presented as mean ± SD. Because human donors produce variable levels of MMP-13 in response to IL-1β/OSM, densitometry data are normalized to IL-1β/OSM and shown as percent inhibition from the positive control. Treatment (mean ± SD): Control (13.23±10.96); IL-1β/OSM (100.0±0.0); PD+IL-1β/OSM (43.13±22.99); SP+IL-1β/OSM (41.18±24.71); SB+IL-1β/OSM (20.07±16.66); Rux+IL-1β/OSM (10.67±5.71). *=p<0.0001

We used the general PI-3 kinase inhibitor LY294002 to determine if inhibition of PI-3 kinase, which is upstream of Akt, would block MMP-13 production. Primary human articular chondrocytes (both normal and OA, data is shown for normal cells) were treated with IGF-1 or cytokines (with or without LY294002) overnight for measurement of MMP levels in conditioned media or for 30 minutes for measurement of Akt phosphorylation. In these experiments, we also examined IL-6 as a stimulus. IGF-1 and the low dose of IL-1β (0.02ng/ml) were not sufficient to increase MMP-13 while OSM and IL-6 did increase MMP-13 which was greatest with OSM (Figure 3A). Levels of MMP-13 in the conditioned media did not correlate with levels of Akt phosphorylation in cell lysates. Akt phosphorylation was strongest with IGF-1 and not detectable with IL-6. However, PI-3 kinase activity was required for the IL-1/OSM mediated increase in MMP-13 since LY294002 blocked MMP-13 production. Inhibition of PI-3 kinase by LY294002 had a similar effect on Akt phosphorylation and MMP-13 production in chondrocytes as in normal cells (data not shown).

### MAP kinase and JAK1/2 inhibition block IL-1β/OSM stimulated MMP-13 production

The disconnect between MMP-13 production and Akt phosphorylation in response to IGF-1 and the cytokines suggests that, although PI-3 kinase activation is required for IL-1β/OSM signaling that results in MMP-13 production, additional signals are also necessary. Since IL-1β/OSM treatment also activates MAP kinase and the JAK/STAT pathway in chondrocytes, we tested the ability of MAP kinase and a JAK inhibitor to block the IL-1β/OSM stimulation of MMP-13. Specific inhibition of ERK, JNK, p38, or JAK1/2 all significantly reduced the amount of IL-1β/OSM stimulated MMP-13 with the greatest reduction noted after inhibition of either p38 or JAK1/2 (Figure 3B and C). Inhibition of JAK1/2 blocked IL-1β/OSM mediated Akt and STAT3 phosphorylation but did not affect IGF-1 mediated Akt phosphorylation. JAK1/2 inhibition also did not affect IL-1β/OSM or IGF-1 mediated ERK, JNK, or p38 phosphorylation (Figure 3D).

### Activity of Akt isoforms 1 and 3 alone is not sufficient to stimulate MMP-13 production

**Figure 4:**
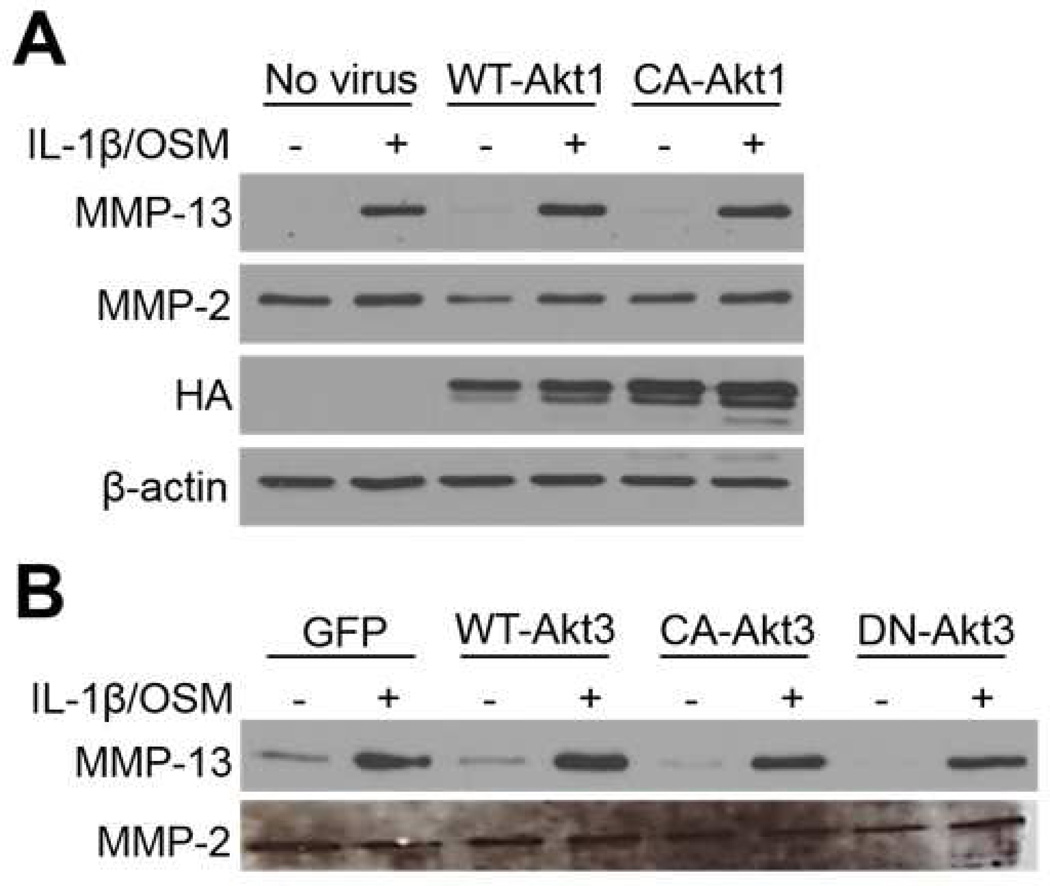
Over-expression of Akt1 or Akt3 has no effect on IL-1β/OSM induced MMP-13 production **A.** Wild type (WT) and constitutively active (CA)-Akt1 were over-expressed in normal chondrocytes, followed by treatment with IL-1β/OSM. Akt 1 constructs contain a hemagglutinin (HA) expression tag, so the HA blot of total lysates is shown to confirm expression of constructs. Representative blots are shown (n=3 independent experiments using cells from three unique tissue donors). **B.** WT, CA, and dominant negative (DN)-Akt3 were over-expressed in normal chondrocytes, followed by treatment with IL-1β/OSM. Representative blots are shown (n=3 independent experiments using cells from three unique tissue donors). MMP-2 is shown as loading control for conditioned media.

In order to further determine the role of Akt activation in chondrocyte MMP-13 production, we utilized a lentivirus construct to over-express wild type or constitutively active Akt1, with or without IL-1/OSM stimulation, and examined MMP-13 production. We had previously shown that chondrocytes transduced with this construct had a marked increase in proteoglycan and collagen synthesis as well as expression of type II procollagen and Sox-9 (5). The overexpression of WT-Akt1 or CA-Akt1 was not sufficient to stimulate MMP-13 production without IL-1β/OSM and did not promote a further increase in MMP-13 levels in cells also treated with IL-1/OSM (Figure 4A). We also tested expression of a dominant negative Akt1 construct but found that the high levels of over-expression needed to compete with endogenous Akt1 resulted in significant cell death (measured by LDH release) such that we could not interpret effects on MMP-13 production (results not shown).

In addition to Akt1, the Akt3 isoform has also been implicated in chondrocyte MMP-13 production (7). We therefore utilized DNA plasmids to over-express wild type, constitutively active, and dominant negative Akt3. The activity of Akt3 alone was not sufficient to increase MMP-13 production, and the amount of MMP-13 stimulated by IL-1β/OSM was not significantly different when WT-, CA- or DN-Akt3 were over-expressed compared to the GFP control (Figure 4B). Unlike our previous findings with constitutively active Akt1 (14), expression of CA-Akt3 did not significantly affect collagen II expression or proteoglycan synthesis (data not shown).

### PI-3K isoforms differentially regulate the chondrocyte response to IL-1β/OSM

**Figure 5:**
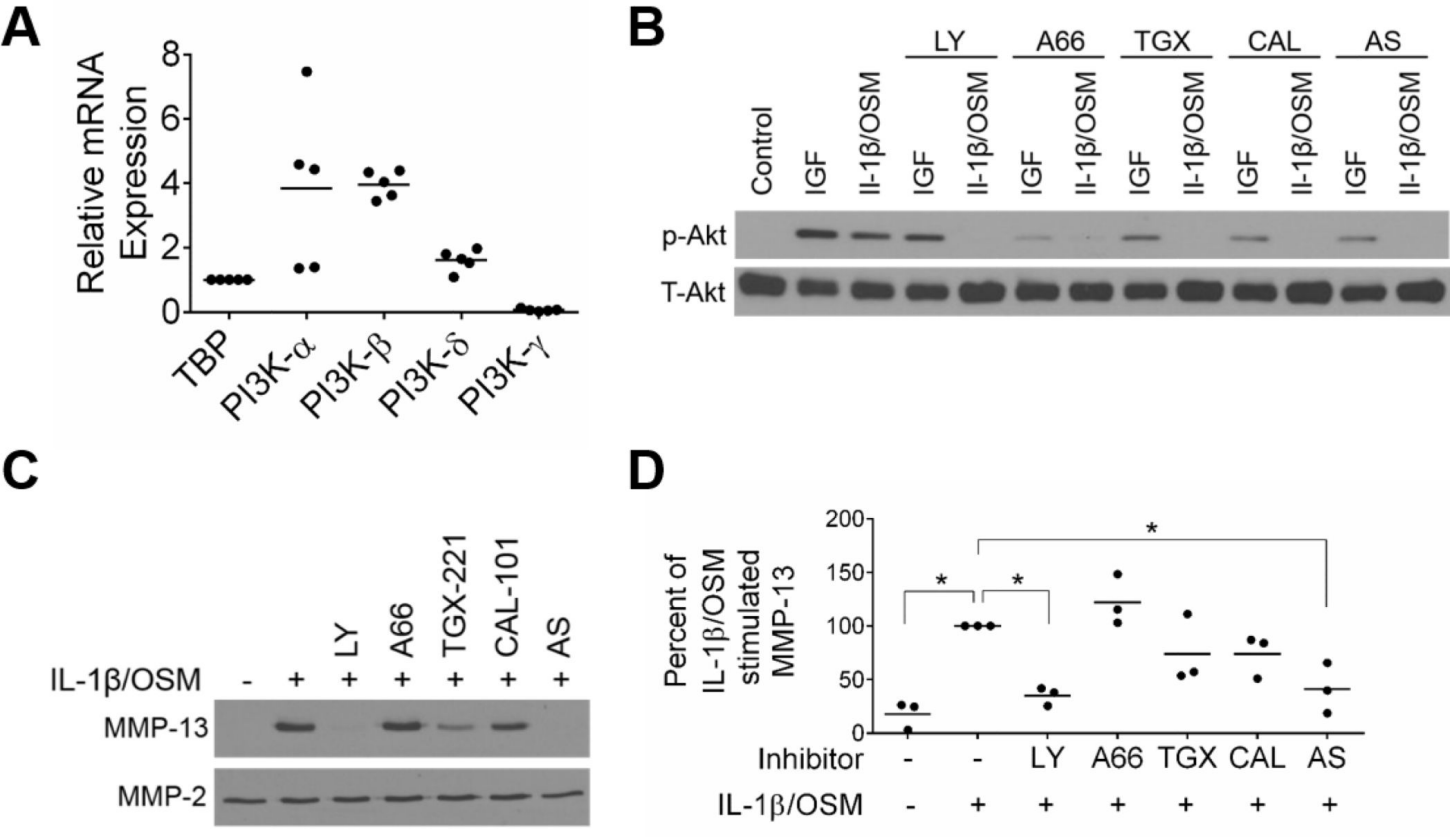
Inhibition of PI-3K-γ significantly reduces IL-1β/OSM induced MMP-13 production **A.** Baseline mRNA expression of PI-3K isoforms in chondrocytes from five normal donors (results are presented as individual data points with the mean indicated by the horizontal line). Expression level (mean±SD): TBP (1.00±0.0); PI-3K-α (3.84±2.56); PI-3K-β (3.97±0.42); PI-3K-δ (1.61±0.34); PI-3K-γ (.06±.04). **B.** Chondrocytes in monolayer were pre-treated with inhibitor (5uM LY or 10uM A66, TGX, CAL, or AS) for 30 min followed by 30 min stimulation with 100ng/ml IGF-1 or 10ng/ml IL-1β+10ng/ml OSM. Representative blots are shown (n=3 independent experiments using cells from three unique tissue donors). **C**. Chondrocytes in monolayer were pre-treated with inhibitors for 30 min followed by overnight stimulation with 10ng/ml IL-1β+10ng/ml OSM. Representative blots from MMP blots of conditioned media are shown (n=3 independent experiments using cells from three unique tissue donors). **D**. MMP-13 in conditioned media was quantified by total MMP-13 ELISA. Data are normalized to amount of MMP-13 stimulated by IL-1β/OSM without inhibitors (set to 100%). Data shown are mean ± SD (n= 3 independent normal tissue donors). Treatment (mean±SD): Control (17.99±13.01); IL-1β/OSM (100.0±0.0); LY+IL-1β/OSM (35.02±8.53); A66+IL-1β/OSM (122.3±23.42); TGX+IL-1β/OSM (73.99±32.16); CAL+IL-1β/OSM (74.00±20.07); AS+IL-1β/OSM (41.38±23.54). Control vs. IL-1β/OSM, p=0.0027; IL-1β/OSM vs. LY+IL-1β/OSM, p=0.176; IL-1β/OSM vs. AS+IL-1β/OSM, p=0.0358.

Multiple isoforms of PI-3 kinase can be expressed in chondrocytes, and so we determined if a specific PI-3 kinase isoform mediated the effects on MMP-13 production in chondrocytes treated with IL-1β and OSM. We first investigated the baseline mRNA expression of these isoforms in chondrocytes and found that PI-3 kinase-α and PI-3 kinase-β were the most highly expressed in chondrocytes. PI-3 kinase-γ and PI-3 kinase-δ were also expressed, but at lower baseline levels than the α and β isoforms (Figure 5A).

We next tested chemical inhibitors specific to each PI-3 kinase isoform. Because the PI-3 kinase-Akt pathway is essential for chondrocyte survival, we confirmed that overnight treatment of chondrocytes with these inhibitors at 10µM did not result in significant cell death (data not shown). Pre-treatment of chondrocytes with the inhibitors followed by IGF-1 or IL-1β/OSM stimulation partially inhibited phosphorylation of Akt (Figure 5B). The 5µM dose of the general PI-3 kinase inhibitor LY294002 tested here was not sufficient to completely block the Akt phosphorylation stimulated by IGF-1 while it did inhibit IL-1β/OSM stimulation of Akt phosphorylation. Previous studies from our group have shown that higher doses of LY294002 (up to 25µM) are required to inhibit IGF-1 mediated Akt phosphorylation, but the lower dose was used in the present study since it was sufficient to block MMP-13 production in response to IL-1β/OSM and would be more directly comparable to the doses of isoform specific inhibitors (4).

We examined the effect of the inhibitors on IL-1β/OSM stimulation of MMP-13 production and found that the inhibitor of PI-3 kinase-γ, AS-252424, reduced the level of IL-1β/OSM stimulated MMP-13 to that seen with the general inhibitor LY294002 (Figure 5C). Inhibition of PI-3 kinase-β with TGX-221 partially inhibited MMP-13 production while the PI-3 kinase-α and –δ inhibitors were the least effective. The inhibitors were further tested using an MMP-13 ELISA which is more quantitative than immunoblotting. The only statistically significant inhibition was seen with the general inhibitor LY294002 and the PI-3 kinase-γ inhibitor AS-252424 (Figure 5D).

## Discussion

Cells such as chondrocytes utilize a complex network of signaling pathways to integrate and appropriately respond to multiple cues in their immediate environment. Diverse stimuli, sometimes even those that mediate opposing functions, can share common signaling proteins and components of particular pathways. The cellular response to such stimuli is determined not by the activity of a single protein or pathway but rather by the integration of multiple signals influenced by both timing and location of the signals within the cell. Here we show that the divergent responses mediated by an anabolic factor, IGF-1, that stimulates cartilage matrix synthesis, and the combination of the catabolic factors IL-1β and OSM, that stimulate cartilage matrix breakdown, share activation of the PI-3 kinase-Akt pathway. The differences among the stimuli which contribute to the divergent responses included the more sustained phosphorylation of Akt and transient phosphorylation of ERK in response to IGF-1 and the additional activation of JNK, p38, and STAT3 by IL-1β and OSM.

The effects of IGF-1, IL-1β, and OSM on chondrocyte signaling have been investigated independently but have not been directly compared (5, 7). The finding that inhibition of PI-3 kinase blocks the increase in MMP-13 is consistent with previous studies (7, 13). It had also been shown that siRNA knock-down of Akt1 or Akt3 inhibits the IL-1β/OSM stimulated MMP-13 production indicating that they are also required for the increase in MMP-13 in response to IL-1β/OSM(7). Our finding that expression of constitutively active Akt1 or Akt3 was not sufficient to increase MMP-13 production shows that although these signaling proteins may be required for the effect of IL-1β/OSM, they are not sufficient by themselves, suggesting additional signaling pathways must also be required.

IL-1β is well known to activate MAP kinase signaling, and OSM can activate the JAK-STAT pathway (11, 12). The ability of the combination of IL-1β and OSM to stimulate chondrocyte MMP-13 production was most dependent on p38 and JAK-STAT activity. The lack of appreciable p38 or STAT3 phosphorylation in response to IGF-1 can explain in part why IGF-1 does not stimulate MMP-13 production despite the strong activation of the PI-3 kinase-Akt pathway.

We also examined the role of specific PI-3 kinase isoforms in MMP-13 production stimulated by IL-1β/OSM. The LY294002 compound that blocked MMP-13 production is very commonly used as a PI-3 kinase inhibitor but is not isoform specific. The most highly expressed isoforms at the RNA level were PI-3 kinase-α and –β followed by PI-3 kinase-δ with little PI-3 kinase-γ. Only inhibition of the γ isoform completely blocked MMP-13 production to the level of LY294002. It is possible that the more highly expressed isoforms were less susceptible to the inhibitors at the doses used, although all of the inhibitors, except A66 for PI-3 kinase-α, had at least a partial effect suggesting there could be some redundancy among the isoforms. Further studies could test knockdown of specific isoforms to confirm the inhibitor results.

**Figure 6:**
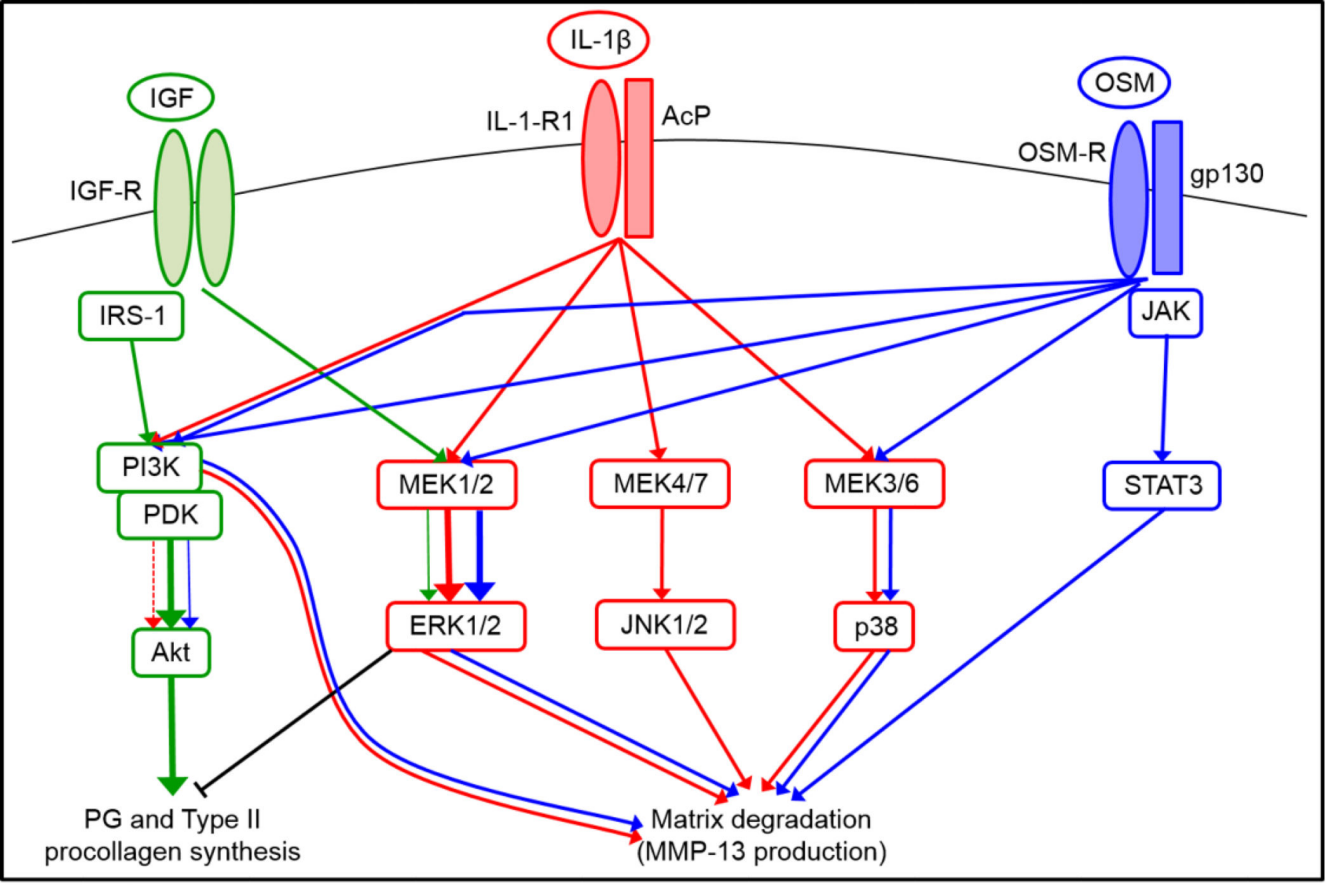
Integrated signaling network of key pathways activated by IGF-1, IL-1β, OSM, and IL-1β/OSM The integrated signaling network is color-coded to track pathways activated by IGF-1 (green), IL-1β (red), OSM (blue), and IL-1β/OSM (parallel red and blue). Solid lines with arrows indicate pathway activation and kinase phosphorylation by indicated stimulus. The dashed line is to indicate that IL-1β by itself does not stimulate Akt phosphorylation but does when used in combination with OSM. The weight of the lines indicates the relative level of phosphorylation stimulated (eg, IGF-1 is a strong stimulator of Akt phosphorylation and PG synthesis, so those green arrows are weighted heavily). This signaling network illustrates that while IGF-1 and the combination of IL-1β and OSM share activation of the PI-3K-Akt pathway, they have different downstream effects due to differential activation of the JNK and p38 MAP kinases as well as STAT3.

Key nodes in the signaling pathways that are activated by IGF-1, IL-1β, and OSM are shown in Figure 6 demonstrating points of overlap and divergence. Previous studies have documented the involvement of individual pathways, such as the IRS-1-PI- kinase-Akt pathway that mediates proteoglycan and collagen production in response to IGF-1 and the MEK-ERK pathway which is inhibitory (4, 5). Of note from the present study, IL-1β alone was not sufficient to phosphorylate Akt or STAT3 while OSM alone was not sufficient to phosphorylate JNK indicating that the combined effect of these cytokines on multiple signaling pathways may be responsible for the observed potent effect of this combination on cartilage matrix destruction (7, 13). The findings from this study suggest that targeting signaling proteins, such as JAK1/2, activated by IL-1β or OSM and not by IGF-1 would provide a better therapeutic approach to restoring the balance in anabolic and catabolic activity in cartilage in order to slow further progression of cartilage loss in OA.

## Figures and Tables

**Table 1:** Summary of inhibitors used in this study.

Inhibitor	IC_50_	Dose (µM)	Inhibitor Target
*LY294002*	1.4µM	5	pan-PI-3 kinase
	
*A66*	32nM	10	PI-3 kinase-α
	
*TGX-101*	10nM	10	PI-3 kinase-β
	
*AS-252424*	33nM	10	PI-3 kinase-γ
	
*CAL-101*	65nM	10	PI-3 kinase-δ
	
*PD98059*	2µM	30	MEK (ERK)
	
*SP600125*	40nM	20	JNK
	
*SB203580*	0.3–0.5µM	10	p38
	
*Ruxolitinib*	3.3/2.8nM	10	JAK1/2 (STAT3)
	
